# Assorted copper pennies on a scar - a case of chromoblastomycosis after knee transplant

**DOI:** 10.1590/0037-8682-0125-2022

**Published:** 2022-07-25

**Authors:** Gopikrishnan Anjaneyan, Radhika Krishna, Jyotsna Yesodharan

**Affiliations:** 1 Amrita Institute of Medical Sciences, Department of Dermatology, Amrita Vishwa Vidyapeetham, Kochi, India.; 2 Amrita Institute of Medical Sciences, Department of Pathology, Amrita Vishwa Vidyapeetham, Kochi, India.

**FIGURE 1:**
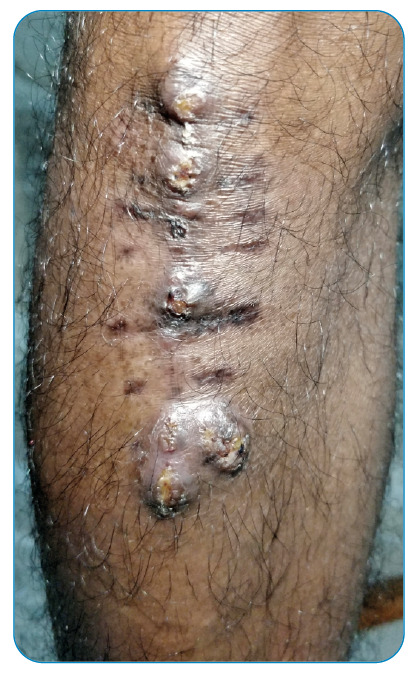
Multiple verrucous plaques with crusting over knee transplant surgery scar.

**FIGURE 2:**
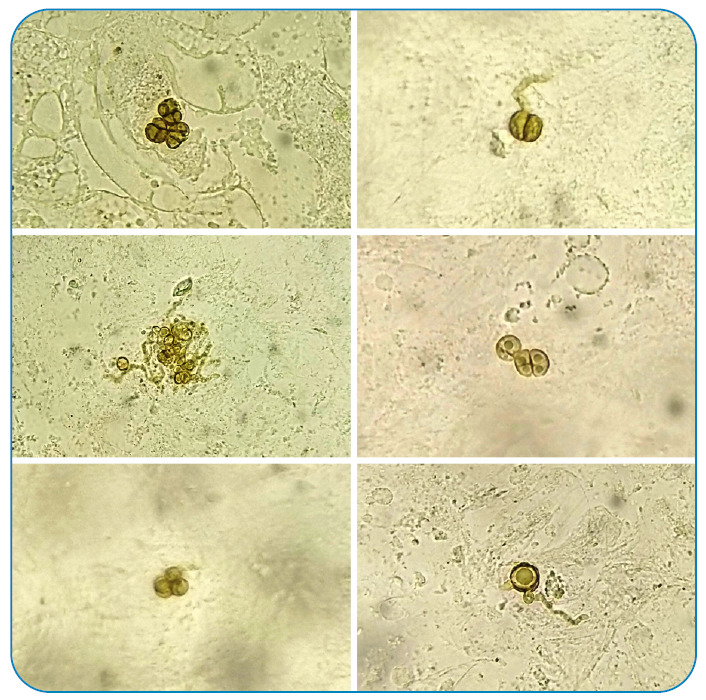
Various morphologies of copper pennies on KOH smear.

**FIGURE 3:**
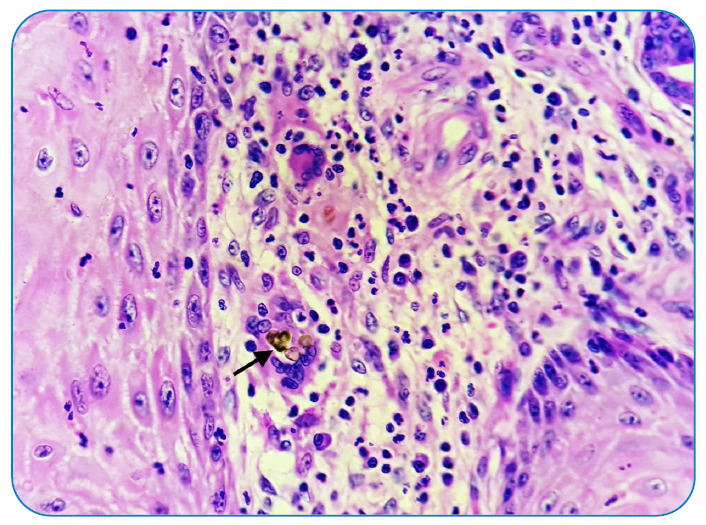
Histopathology showing copper penny bodies of chromoblastomycosis (Hematoxylin & Eosin, 400x).

A 44-year-old man who had undergone renal transplantation and on immunosuppressants (tacrolimus) presented with asymptomatic thick hyperpigmented lesions over the right knee for one month (Figure 1). He underwent knee transplant surgery 1.5 months back, after which the lesions started and progressively increased in size. Local examination revealed multiple well-defined skin-colored to hyperpigmented verrucous plaques and nodules with superficial crusts over the right knee at the scar site. Skin scraping with 10% potassium hydroxide (KOH) showed multiple round thick-walled brownish budding bodies resembling different morphologies of copper pennies (also known as sclerotic/muriform/medlar bodies) (Figure 2). An incision biopsy of the lesion showed pseudoepitheliomatous hyperplasia with dermal suppurative granulomas and copper penny bodies, suggestive of chromoblastomycosis (Figure 3). After discussion with the treating nephrologist, he was started on treatment with itraconazole 100 mg twice daily along with cryotherapy^1^, following which the lesions started to improve, and he is currently undergoing regular follow-up.

Chromoblastomycosis, a chronic subcutaneous fungal infection, is caused by pigmented fungi such as *Phialophora verrucosa, Fonsecaea pedrosoi, Fonsecaea compacta,* and *Cladophialophora carrionii* ^2^. Combined histopathological and mycological diagnosis, including a KOH smear, is a highly sensitive approach^3^. An interesting feature noted in our case was the various unique morphologies of the copper pennies identified on the KOH smear. This highlights that a simple and inexpensive office procedure can allow timely diagnosis and early treatment of this subcutaneous mycosis, thereby preventing subsequent complications. 
